# COVID-19 increases extracorporeal coagulation during hemodialysis associated with upregulation of vWF/FBLN5 signaling in patients with severe/critical symptoms

**DOI:** 10.1186/s12879-024-09245-9

**Published:** 2024-04-22

**Authors:** Guang Yang, Hui Shan, Dibin Wu, Sanmu Li, Zhiwei Lai, Fengping Zheng, Zibo Xiong, Zuying Xiong, Yuhan Diao, Ying Shan, Yun Chen, Aihong Wang, Wei Liang, Yuxin Yin

**Affiliations:** 1 Division of Renal Medicine, Peking University Shenzhen Hospital, Peking University, Shenzhen, 518036 China; 2 Shenzhen Clinical Research Centre for Urology and Nephrology, Shenzhen, 518036 China; 3 Institute of Nephrology, Shenzhen Peking University-Hong Kong University of Science and Technology (PKU-HKUST) Medical Center, Shenzhen, 518036 China; 4 Precision Medicine Research Institute, Peking University Shenzhen Hospital, Peking University, Shenzhen, 518036 China; 5 Department of Medical Records & Statistics, Peking University Shenzhen Hospital, Peking University, Shenzhen, 518036 China; 6 Clinical Research Academy, Peking University Shenzhen Hospital, Peking University, Shenzhen, 518036 China; 7 Institute of Ultrasound Medicine, Shenzhen Peking University-Hong Kong University of Science and Technology (PKU-HKUST) Medical Center, Shenzhen, 518036 China

**Keywords:** Anticoagulant, Blood clots, Coagulation, Hemodialysis, New coronavirus-19

## Abstract

**Background:**

COVID-19 has been shown to increase the risk of extracorporeal coagulation during hemodialysis in patients, but the underlying mechanism remains unclear. This study aimed to investigate the effect and mechanism of COVID-19 on the risk of extracorporeal coagulation in patients with chronic kidney disease undergoing hemodialysis.

**Methods:**

A retrospective analysis of the extracorporeal coagulation status of 339 hemodialysis patients at our center before and after COVID-19 infection was performed, including subgroup analyses. Post-infection blood composition was analyzed by protein spectrometry and ELISA.

**Results:**

Compared to the pre-COVID-19 infection period, COVID-19-induced extracorporeal coagulation predominantly occurred in patients with severe/critical symptoms. Further proteomic analysis demonstrated that in patients with severe/critical symptoms, the coagulation cascade reaction, platelet activation, inflammation, and oxidative stress-related pathways were significantly amplified compared to those in patients with no/mild symptoms. Notably, the vWF/FBLN5 pathway, which is associated with inflammation, vascular injury, and coagulation, was significantly upregulated.

**Conclusions:**

Patients with severe/critical COVID-19 symptoms are at a higher risk of extracorporeal coagulation during hemodialysis, which is associated with the upregulation of the vWF/FBLN5 signaling pathway. These findings highlight the importance of early anticoagulant therapy initiation in COVID-19 patients with severe/critical symptoms, particularly those undergoing hemodialysis. Additionally, vWF/FBLN5 upregulation may be a novel mechanism for virus-associated thrombosis/coagulation.

**Supplementary Information:**

The online version contains supplementary material available at 10.1186/s12879-024-09245-9.

## Introduction

Patients on maintenance hemodialysis (HD), who typically suffer from advanced chronic kidney disease (CKD) or even end-stage kidney disease (ESKD), require hemodialysis 2–4 times weekly to maintain blood homeostasis and normal physiological function [1, 2]. Extracorporeal coagulation is a common acute complication during hemodialysis treatment, impacting both dialysis efficiency and patient safety [3]. This is because impaired kidneys may lead to a greater higher risk of bleeding or clotting through various factors, such as platelet dysfunctions, uremic toxins, disrupted hemostasis, endothelial dysfunction, and inflammation [4, 5]. In most cases, CKD patients exhibit an elevated risk of coagulation dysfunctions, characterized by heightened levels of D-dimer, TAT, and ICAM-1 but decreased levels of the platelet aggregation biomarkers adenosine diphosphate and thrombin receptor-activating peptide [4]. Therefore, monitoring extracorporeal coagulation and taking therapeutic measures during hemodialysis are critical for ensuring patient safety.

Coronavirus disease-19 (COVID-19) not only exacerbates the risk of thrombosis in vivo, such as deep vein thrombosis, pulmonary embolism, and stroke [6–8], but also increases the risk of extracorporeal circuits during hemodialysis treatment [9–11]. The virus is thought to trigger inflammatory responses, increase factor VIII levels, and prolong the activity of coagulation factors, resulting in coagulation abnormalities [12–14]. Some patients with severe COVID-19 have also been found to have elevated levels of fibrinogen and D-dimer, proteins that are markers of blood clotting [13]. To prevent thrombosis, anticoagulants are commonly used for treating some COVID-19 patients [15, 16]. However, it remains unclear whether the cause of COVID-19-induced coagulation abnormalities is consistent across the general and CKD patient populations.

COVID-19 is just one example of an infectious disease, and there may be future outbreaks of COVID-19 viral variants or other pathogens [17]. For example, two additional large-scale outbreaks of COVID-19 mutations occurred in China in July 2023 and December 2023, coinciding with a noticeable rise in dialysis-associated extracorporeal coagulation. Drawing from this experience, hospital staff implemented various anticoagulant therapies promptly to prevent dialysis failure and potential hazardous scenarios. Therefore, this study aimed to analyze the effect of COVID-19 on extracorporeal coagulation risk during hemodialysis and explore underlying mechanisms. This study was divided into two parts: (1) retrospective analysis of the COVID-19's effect on extracorporeal coagulation risk and (2) analysis of protein profiles in patients with severe/critical symptoms to identify the causes of coagulation dysfunctions. Importantly, the COVID-19 restrictions were lifted by the government on December 5, 2022, which was a unique period of study, with all patients exhibiting marked symptoms of infection, in contrast to their prior infection-free status, making this investigation particularly representative.

## Methods

### Ethics

This study collected clinical data, blood samples, and imaging data from patients in a manner that did not interfere with their treatment protocols and posed no risks to them. All patients signed an informed consent form. This study adhered to the Declaration of Helsinki and received approval from the Clinical Research Ethics Committee of Peking University Shenzhen Hospital for its relevant research (No. 2023–088).

### Patients

Patients who were undergoing hemodialysis treatment at the Hemodialysis Center of Peking University Shenzhen Hospital between December 1, 2022 and February 28, 2023 were selected for the present study. The information of 339 patients, including 224 males and 115 females, was included in the final analysis. It is crucial to highlight that our study implemented rigorous controls, including daily nucleic acid testing for all patients. Prior to December 5, 2022, none of the patients tested positive for COVID-19. However, following the relaxation of restrictive regulations, a majority of patients exhibited notable COVID-19 symptoms within one week, which were subsequently confirmed through nucleic acid testing.

The inclusion criteria were as follows: (i) CKD patients who were on maintenance hemodialysis for ≥ 3 months; and (ii) had COVID-19 infection confirmed by nucleic acid testing. The exclusion criteria were as follows: (i) Dialysis patients not infected with COVID-19; (ii) Insufficient blood flow due to vascular access dysfunction leading to coagulation in filters and lines; (iii) Coagulation in filters and lines due to the use of blood products during dialysis treatment; (iv) Coagulation due to heparin-free treatment; (v) Infected with other pathogens; (vi) A family history of coagulation dysfunction, especially in patients with known or suspected hereditary thrombophilia; (vii) According to the vaccination recommendations, all patients with cardiovascular-related underlying diseases had not received any vaccine. Therefore, vaccinated patients were excluded from this study.

### Severity criteria

COVID-19 infection severity criteria were based on the latest "Diagnosis and treatment protocol for COVID‐19 patients (Trial Version 10)" [18]. (i) Mild: Upper respiratory tract infection is the primary symptom, presenting with dry throat, sore throat, cough, fever, and similar symptoms. (ii) Moderate: Symptoms persist for more than three days, accompanied by a cough, shortness of breath, and other symptoms. The respiratory rate remains below 30 breaths times per minute, and the resting-state finger oxygen saturation is above 93%. Imaging reveals manifestations of COVID-19 infection pneumonia. (iii) Severe: Shortness of breath is present, with a resting respiratory rate above 30 breaths per minute. The SPO_2_ concentration decreases to less than 93% after air inhalation, oxygenation index PaO_2_/FiO_2_ is below 300 mmHg, and the patient’s clinical symptoms and lung imaging results progressively worsen, with significant lesions exceeding 50% within 24–48 h. (iv) Critical: Respiratory failure occurs, necessitating mechanical ventilation; shock is present; and other organ failures require ICU monitoring and treatment.

### Hemodialysis

Following infection with COVID-19, patients adhere to a prescribed regimen of 2–4 dialysis sessions per week to complete their treatment. The standard protocol for hemodialysis is employed for patients who possess stable vital signs and no substantial reduction in blood oxygen levels. Treatment requires a 4-h duration, a blood flow of 200–300 mL/min, and dialysate flow of 500–600 mL/min. This procedure follows the operating procedures of the 2021 SOP standards and uses the same technical operations as the treatment protocol implemented prior to contracting the virus.

Continuous renal replacement therapy (CRRT) treatment protocols are designed for patients in severe or critical phases of COVID-19 infection who are experiencing a significant decrease in blood oxygen levels, respiratory and circulatory problems, and multi-organ failure that cannot be treated with hemodialysis. These protocols are developed collaboratively by physicians at the hemodialysis center and are customized for individual patients based on their specific conditions. The treatment employs an autologous arteriovenous endovascular fistula or an intravenous catheter for vascular access and primarily utilizes the continuous venovenous hemodiafiltration method. The treatment duration is typically set between 6–10 h, with replacement fluid containing glucose (10.6 mmol/L), chloride (118 mmol/L), magnesium (0.797 mmol/L), calcium (1.6 mmol/L), sodium (113 mmol/L), and potassium (adjusted with timing) concentrations. The blood flow is kept between 180–220 mL/min, and the replacement fluid is set at 2000–3000 mL/h, and dilution before replacement fluid is given is set greater than 50%. To ensure the efficacy of the treatment, biochemical indicators such as creatinine, urea nitrogen, potassium, sodium, calcium, chloride, and the nitrogen dioxide binding rate were monitored hourly.

Most patients undergo heparin administration before hemodialysis, a standard procedure guided by previous clinical practice. Heparin is delivered via the arterial end of the vascular access system at a prescribed dosage of 5,000 IU (60–80 IU/kg). In cases of severe coagulation dysfunction, the dosage may be adjusted (up to 2X for hemodialysis and 3X for CRRT), with potential consideration for concurrent regional citrate anticoagulation. Notably, only one patient encountered anticoagulation treatment failure but successfully recovered after transitioning to peritoneal dialysis.

### Instrument monitoring

During hemodialysis and CRRT treatment, blood flow, venous pressure, arterial pressure, and transmural pressure changes were recorded hourly, along with observations of vital signs and color changes in the filter and arteriovenous port of the bloodline tube. If severe clotting blockage is detected, treatment should be discontinued early. In cases where the treatment goal has not been achieved due to severe clotting blockage, the filter and line should be replaced, and the anticoagulation scheme should be adjusted to restart the treatment. Clotting blockage severity was assessed at the end of the treatment and is categorized as Grade-0 (no clotting), Grade-I (mild clotting), Grade-II (moderate clotting), or Grade-III (severe clotting). The dialysis coagulation grade was based on the Standard Manual of Hemodialysis Treatment [19]: Grade-0, no coagulation, Grade-I: < 10% hollow fiber coagulation, Grade-II < 50% hollow fiber coagulation, and Grade-III: > 50% hollow fiber coagulation. The information and filter lifespan during treatment for COVID-19 patients were extracted from the electronic medical records and compared with those from patients receiving dialysis prior to COVID-19 infection.

### Blood collection

The blood used for analysis was isolated from the residual sample of routine blood tests. Heparin is injected prior to hemodialysis, did not affect protein profile. Briefly, venous blood was collected from the elbow and stored in an anticoagulation tube (#106,680,720, EDTA-K2-2.0 mg/mL, Ivory, China), followed by centrifugation at 3000 RPM for 10 min. Ten microliters of plasma was collected, and the virus was inactivated at 56°C for 30 min. A mixture was prepared by adding alcohol at a 1:1 ratio, and 1% PMSF was subsequently added after evaporation. The sample was preserved at -80°C for subsequent protein profiling (Thermo Fisher Orbitrap Exploris 480).

### Sample preparation

Three microliters of plasma from each specimen was denatured in 30 μL buffer containing 8 M urea in 100 mM a triethylammonium bicarbonate (TEAB) at 32 ℃ for 30 min. The proteins were reduced with 10 mM tris (2-carboxyethyl) phosphine (TCEP) for 30 min at 32 ℃, and then alkylated with 40 mM iodoacetamide (IAA) in darkness at room temperature (25 ℃) for 45 min. The protein extracts were diluted with 220 μL of 100 mM TEAB, and 1–2 μg of trypsin (Promega) was added to each filter. The samples were incubated overnight at 37 °C and the reaction was stopped by adding 30 μL of 10% trifluoroacetic acid (TFA) in volume. The samples were spun at 20,000 g at 4 °C for 10 min. The peptides were desalted using Oasis HLB 1 cc Vac Cartridge columns from Waters according to the manufacturer's instructions. After drying, the peptides were resuspended in 0.1% formic acid.

### LC–MS/MS analysis

Nano-LC–MS/MS analysis was conducted using a Thermo Scientific Orbitrap Exploris 480 instrument equipped with a nano ultra-performance liquid chromatograph (UPLC) (Thermo Scientific Ultimate 3000). Peptides were trapped on a trapping column (Acclaim™ PepMap™ 100 C18 HPLC, 75 µm × 2 cm, Thermo Scientific) and separated on an analytical column (Acclaim™ PepMap™ 100 C18 HPLC, 75 µm × 25 cm, Thermo Scientific). We loaded 1µg onto the column and separation was achieved using a 120 min gradient of 3 to 90% ACN in 0.1% FA at a flow rate of 300 nL/min. Both precursor and fragment ions were acquired in the Orbitrap mass analyzer. The RF lens frequency was set to 40%, the spray voltage was set to 2.5 kV and the ion transfer tube temperature was set to 320 °C. The full scan was performed between 350–1,200 m/z at 120,000 resolution (AGC target of 1 × 106 or 50 ms injection time). Then, 80 DIA segments were acquired at 30,000 resolution with an AGC target of 1 × 106 and 50 ms for maximal injection time. HCD fragmentation was set to normalized collision energies of 25, 30, and 35%. The default charge state for the MS2 was set to 3. The mass spectrometry proteomics data have been deposited in the ProteomeXchange via the iProX partner repository with the dataset identifier PXDO44377 [20, 21].

### Mass spectrometric data analysis

The DIA data were analyzed with Spectronaut 17, a mass spectrometer vendor-independent software from Biognosys. The default settings were used for the Spectronaut search. Decoy generation was set to scrambled (no decoy limit). Interference correction at the MS2 level was enabled. The false discovery rate (FDR) was set to 1% at the peptide level. We used the 'Wu Kong' platform (https://www.omicsolution.com/wkomics/main/) for relative statistical and functional analysis. The Reactome analysis was performed according to the instructions on the website (https://reactome.org).

### Protein level

Relative protein expression levels were determined using the raw PG quantity (already normalized) derived from the mass spectrometry results, and subsequently validated through enzyme linked immunosorbent assay (ELISA). Fibulin-5 (FBLN5, FY-EH7477, Feiyuebio, China) and von Willebrand factor (vWF, JM1008, Jingmei, China) kits were obtained from a third-party commercial supplier, and the experimental procedures were conducted according to the provided instructions.

### Statistics

The data are presented as mean ± standard deviation (SD). Statistical analysis was conducted using GraphPad Prism V6.0. The differences between the two groups were analyzed using an unpaired Student's T-test. Differences in coagulation between patients with different symptoms were analyzed using the Pearson's Chi-square test. A *p-value* ≤ 0.05 was considered to indicate statistical significance.

## Results

### COVID-19 increased the prevalence of extracorporeal coagulation during hemodialysis

**Fig. 1:**
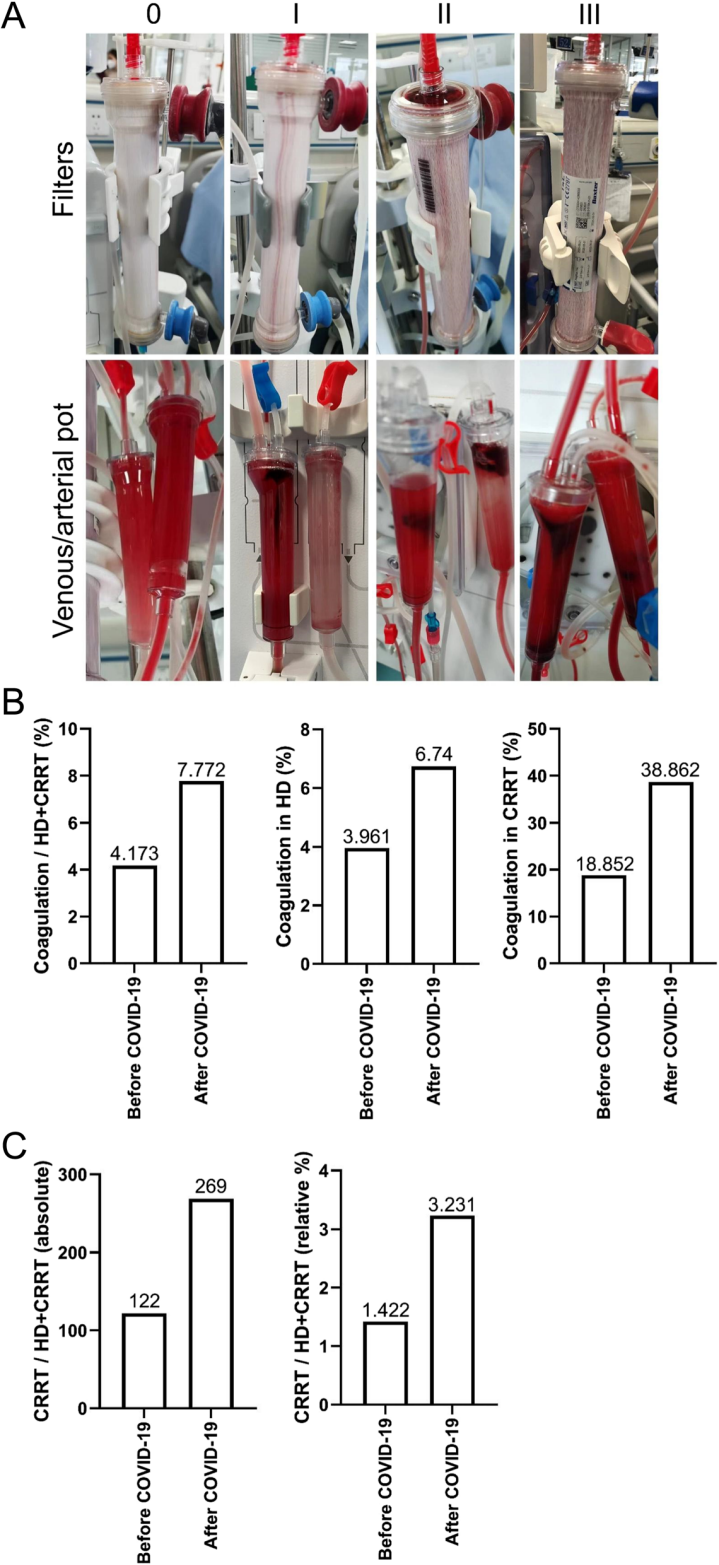
COVID-19 increased the prevalence of extracorporeal coagulation during hemodialysis. **A** Representative images of the different grades of extracorporeal coagulation of the hemodialysis machine. **B** Incidence of coagulation before and after the COVID-19 outbreak. "Before" refers to the period from October to November 2022, and "after" refers to the period from December 2022 to January 2023. **C** The demand for CRRT. HD, hemodialysis. CRRT, continuous renal replacement therapy

To investigate the relationship between COVID-19 and extracorporeal coagulation events, all coagulation events that occurred during hemodialysis were recorded. Images of COVID-19-induced extracorporeal coagulation during hemodialysis were taken. As depicted in Fig. 1A, the filters and venous/arterial pots experienced more severe coagulation as the degree of coagulation increased. In the absence of coagulation (Grade-0), the filters had almost no clots. However, in Grade-I and -II patients, there was slight coagulation in the filters and significant clots in the venous/arterial pots. When Grade III was attained, most of the filters had lost functionality, and consumables had to be replaced.

Subsequently, the coagulation incidence rate was analyzed both before and after COVID-19 infection. As illustrated in Fig. 1B, the incidence of coagulation in all hemodialysis treatments significantly increased during the COVID-19 outbreak. Specifically, the likelihood of coagulation during regular hemodialysis increased from 3.961% to 6.74%, whereas the probability of coagulation during CRRT increased from 18.852% to 38.862%. Moreover, as depicted in Fig. 1C, the use of CRRT escalated from 122 (1.422%) to 269 (3.231%), indicating a greater demand for a smoother hemodialysis modality.

### Severe/critical symptoms of COVID-19 increase the incidence of extracorporeal coagulation

**Fig. 2:**
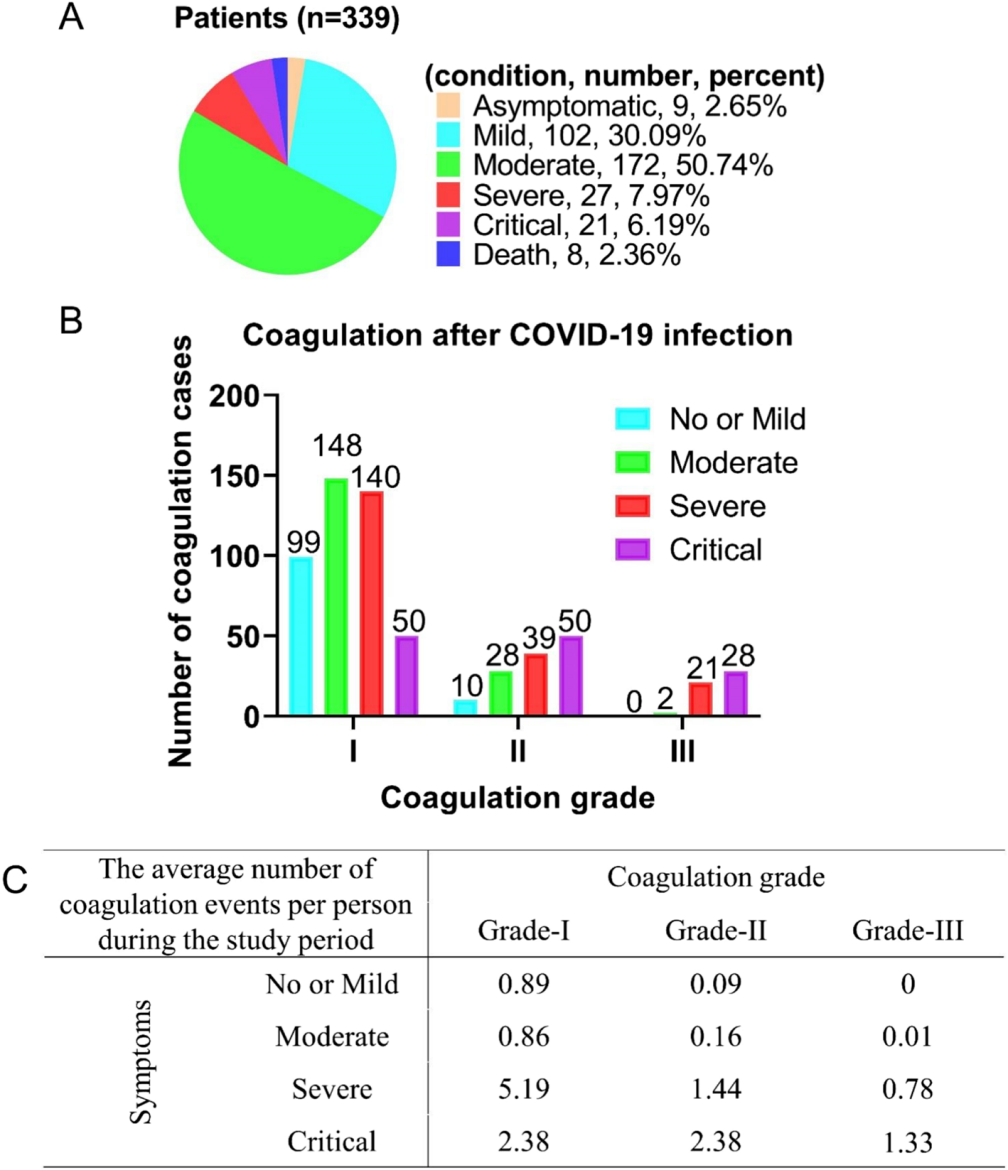
Relationship between coagulation grade and disease severity. **A** Number and proportion of patients with different symptom statuses. **B** The number of coagulations occurring in patients with different symptoms at different coagulation levels. **C** The average number of coagulation events per person during the study period. Pearson's Chi-square test (*p* ≤ 0.05)

In practice, we found that patients with severe COVID-19 symptoms are more likely to experience extracorporeal coagulation events. To elucidate the correlation between COVID-19 symptoms and coagulation grade, the severity of symptoms and coagulation grade of all patients following the COVID-19 outbreak were quantified. The study cohort consisted of 339 patients, including 9 asymptomatic, 102 mild, 172 moderate, 27 severe, 21 critical, and eight deceased patienst (Fig. 2A). Next, this study presents an initial analysis of the association between COVID-19 symptoms and coagulation grade. Among patients with no/mild, moderate, severe, and critical symptoms, the occurrences of Grade-I coagulation were 99, 148, 140, and 50 times, Grade-II coagulation were 10, 28, 39, and 50 times, and Grade-III coagulation were 0, 2, 21, and 28 times, respectively (Fig. 2B). Notably, there was a noticeable increase in the proportion of severe/critical patients among those with Grade-II and -III coagulation, while this trend was not apparent in Grade-I. This observation suggests a potential correlation between the likelihood and severity of extracorporeal coagulation and the severity of the COVID-19 symptoms.

Subsequently, the average number of coagulation occurrence per person was calculated for patients with different symptoms during the study period (Fig. 2C). The results revealed that the average times of Grade-I, -II, and -III in patients with no/mild symptoms were 0.89, 0.09, and 0, respectively. For patients with moderate symptoms, the average durations were 0.86, 0.16, and 0.01 for Grade-I, -II, and -III, respectively. For patients with severe symptoms, the average times were 5.19, 1.44, and 0.78 for Grade-I, -II, and -III, respectively. Finally, for patients with critical symptoms, the average times were 2.38, 2.38, and 1.33 for Grade-I, -II, and -III, respectively. Interestingly, when no or mild symptoms were utilized as the control group, moderate symptoms exhibited minimal to no increase in the incidence of coagulation. However, a notable contrast was observed in patients with severe and critical symptoms, where the likelihood of coagulation at all grades substantially increased. Critical symptoms were found to be more closely associated with Grade-II and -III coagulation than severe symptoms. Finally, Pearson's Chi-square test was used to test the relationship between COVID-19 symptoms and coagulation. The results showed a strong correlation (*p* ≤ 0.05). These findings suggest that severe/critical symptoms of COVID-19 are more likely to trigger extracorporeal coagulation, with a greater probability of coagulation as symptom severity intensifies.

### Significant differences in blood composition between severe/critical and no/mild symptomatic patients

**Fig. 3:**
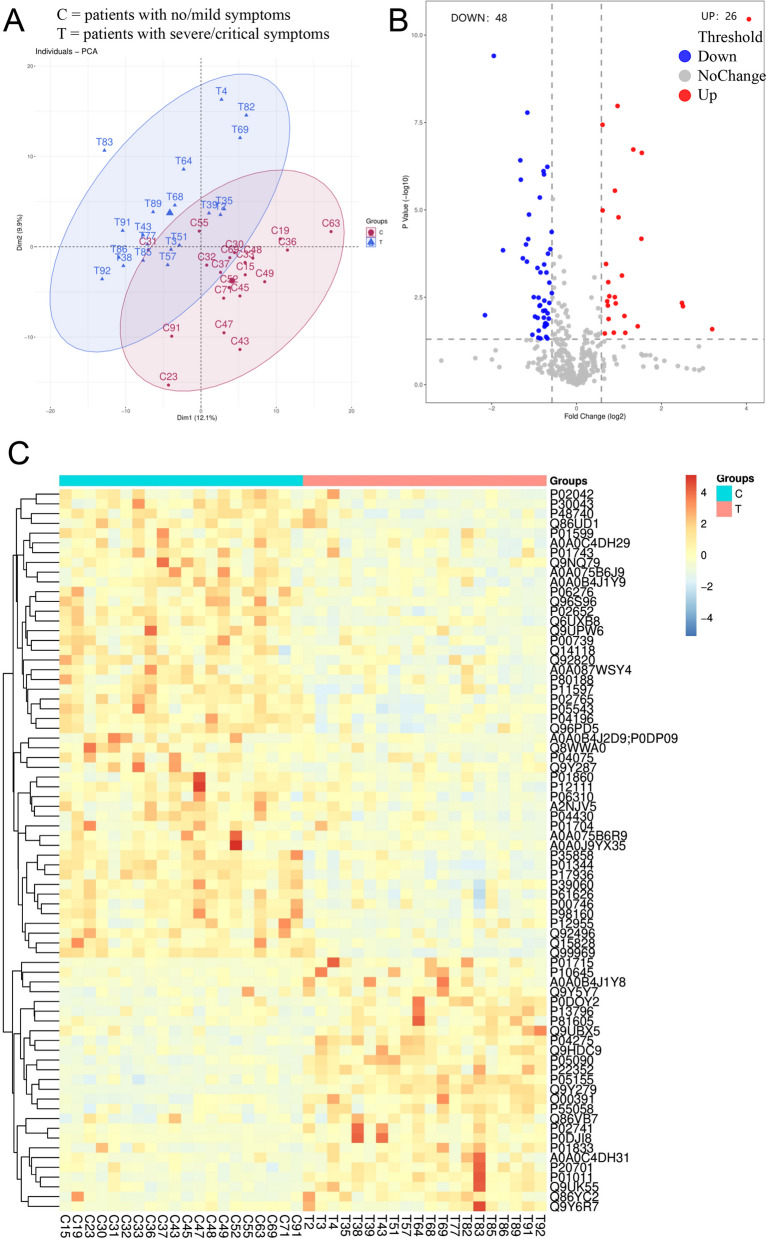
Differences in blood composition between no/mildly symptomatic patients and severe/critical patients. **A** Principal component analysis (PCA) illustrated that there were differences in the components of the two patient groups. **B** The volcano plot showing 26 proteins that were significantly up-regulated and 48 proteins that were significantly down-regulated. **C** Heat map showing differential protein layer clustering analysis. C = patients with no/mild symptoms. T = patients with severe/critical symptoms

To understand the reason for severe/critical symptom-induced extracorporeal coagulation in COVID-19 patients, we selected 20 no/mild symptomatic and 20 severe/critical symptomatic patients and collected their plasma samples for proteomic profiling. To avoid the influence of the transition group on the outcomes, data from the moderate symptom group were excluded from the study analysis. The results showed significant differences in plasma composition between the two groups (Fig. 3A). Compared to the no/mild symptomatic patients, 48 proteins were down-regulated, and 26 proteins were up-regulated in the plasma of severe/critical symptomatic patients (Fig. 3BC).

### Patients with severe/critical symptoms have more coagulation-related factors in their blood

**Fig. 4:**
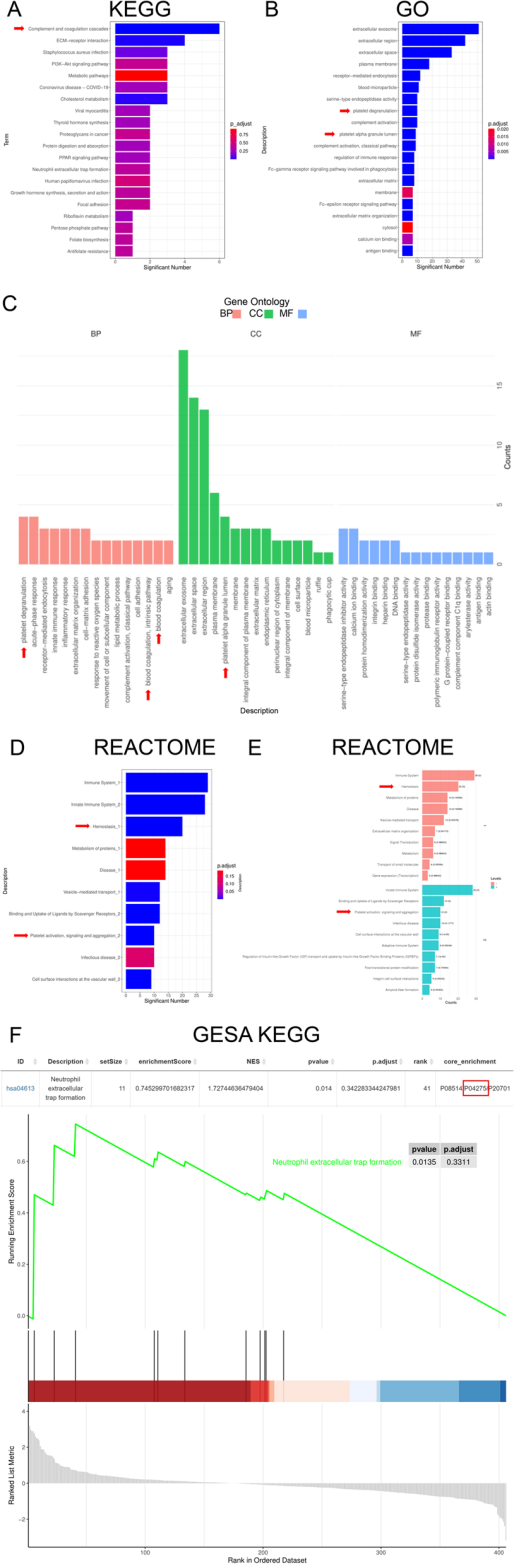
Biological ramifications of variations in blood components. **A** KEGG analysis revealed a significant activation of complement and coagulation-related pathways in patients with severe/critical symptoms. **B** GO analysis uncovered significant abnormalities in the extracellular matrix, cell membrane function, and platelet-related pathways in patients with severe/critical symptoms. **C** Provides a detailed breakdown of the GO analysis, primarily categorized into biological process (BP), cellular component (CC), and molecular function (MF) categories. **D** Reactome analysis identified significant abnormalities in immune responses, hemostasis, platelet, extracellular matrix, cell membrane function, and vascular wall-related pathways in patients with severe/critical symptoms. **E** Offers a detailed explanation of the Reactome analysis. **F** GSEA offers a further interpretation of the KEGG analysis for a single pathway. Multiple pathway analyses were focused on vWF, and the most representative GSEA analysis is presented here

To gain insights into the potential biological mechanisms of these variations in blood components, we conducted further analysis using bioinformatics tools. Kyoto Encyclopedia of Genes and Genomes (KEGG) and Gene Ontology (GO) analyses demonstrated that COVID-19 promoted coagulation cascade reactions, platelet activation, inflammation, and oxidative stress-related signaling pathways (Fig. 4A, B, C). Consistently, differential protein Reactome fractionation enrichment analysis demonstrated that COVID-19 perturbs blood homeostasis and triggers platelet activation (Fig. 4D, E). Our supplementary analyses corroborated these findings. Notably, KEGG analysis of all upregulated proteins revealed that the activation of coagulation-related signals was the main notable difference (Figure S1 & S2). Furthermore, gene set enrichment analysis (GSEA) KEGG enrichment analysis of the entire protein dataset highlighted significant disparities in neutrophil extracellular trap formation, with particular attention given to the P04275 (vWF) protein as a pivotal regulator of coagulation (Fig. 4F). Collectively, our findings strongly suggest that COVID-19 exacerbation markedly upregulates the expression of signaling pathways linked to coagulation.

### COVID-19 upregulated the vWF/FBLN5 pathway

**Fig. 5:**
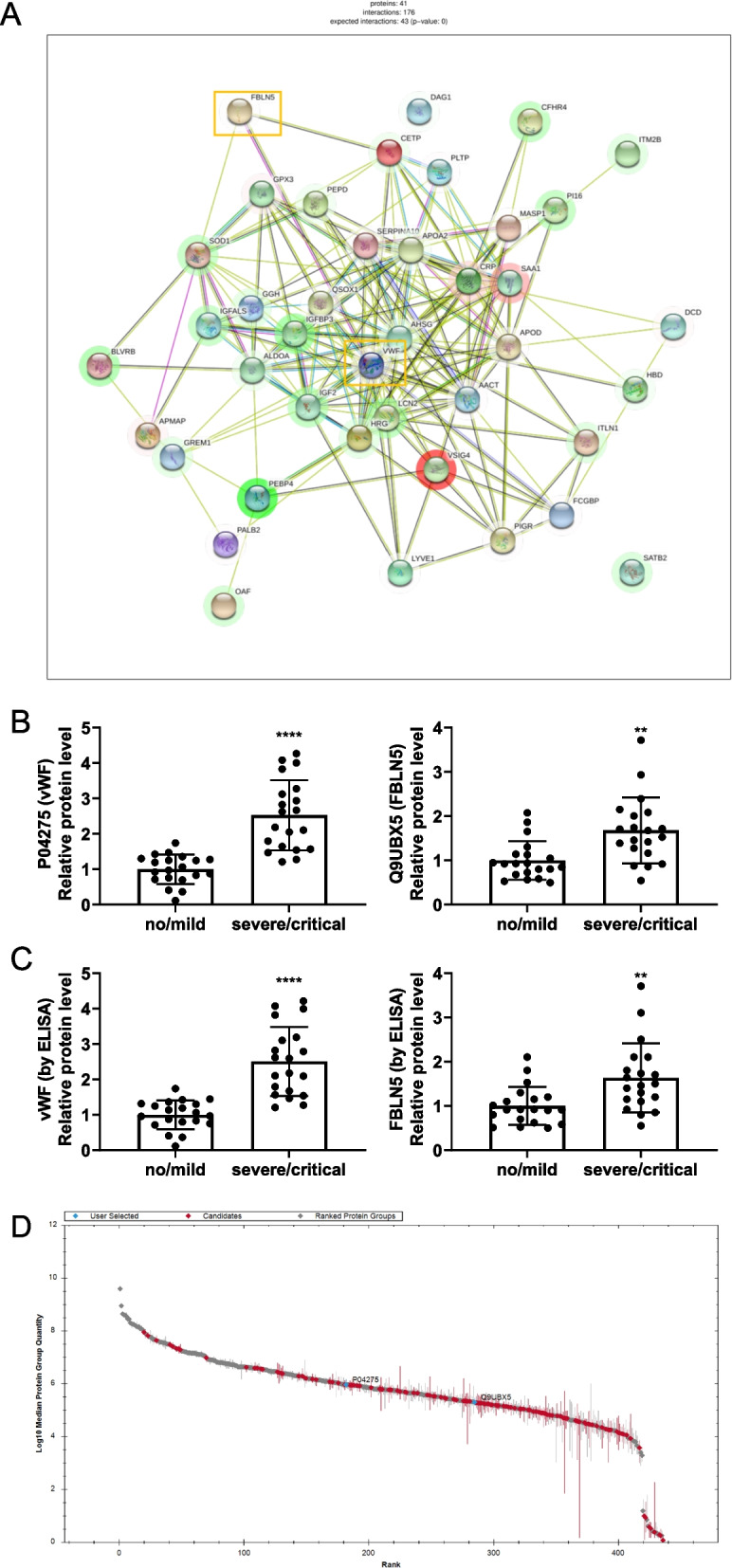
Interaction analysis of COVID-19-related proteins. **A** PPI network analysis. **B** Relative protein expression levels of vWF and FBLN5. **C** Relative protein expression levels of vWF and FBLN5 verified by ELISA. **D** Range map of differential protein kurtosis. **, *p* ≤ 0.01. ****, *p* ≤ 0.001

To explore the pivotal signaling pathways underlying COVID-19-induced extracorporeal coagulation, we employed protein–protein interaction (PPI) network analysis to identify the proteins that exhibit key regulatory functions (Fig. 5A). Subsequent screening revealed that the procoagulant-related protein von vWF plays a crucial role in network regulation along with FBLN5, a protein closely associated with thrombosis. Notably, PPI analysis demonstrated that these proteins act directly in a linear manner. The relative expression of these genes was further evaluated, and the results revealed that the expression of vWF and FBLN5 was significantly upregulated in patients with severe/critical symptoms compared to those with no/mild symptoms (Fig. 5B, D). To corroborate this finding, it was also validated using ELISA (Fig. 5C). The ELISA results exhibited a consistent trend with the protein profiling results, indicating not only a notable disparity in vWF and FBLN5 expression but also confirming the precision of the protein profiling. Collectively, these results suggest that the vWF/FBLN5 pathway may play a pivotal role in the regulation of COVID-19-induced extracorporeal coagulation.

## Discussion

COVID-19 is characterized by an incredibly high transmission rate, with 90.2% of the hemodialysis population being infected within a month of the initial outbreak at our hemodialysis center. This caused extensive thrombotic phenomena that exacerbated the extracorporeal coagulation disruption during hemodialysis and increased the ensuing complications and mortality. The central finding of this study is that COVID-19 infection exacerbates the extracorporeal coagulation level of CKD patients in a symptom’s severity-dependent manner, especially in the patients with severe/critical symptoms, and that this blood hypercoagulable state is associated with the upregulation of vWF/FBLN5 signaling. This finding explains not only the vulnerability of COVID-19 patients to coagulation on hemodialysis, but also their vulnerability to thrombotic death. This study provides a basis for the future management of related complications.

In contrast to other investigations, the present study adopted a more targeted approach to data collection. The rigorous management of the epidemic by the Chinese Government effectively prevented any of the patients at our hemodialysis center from contracting COVID-19 prior to the commencement of the study. On December 5, 2022, the epidemic was deregulated in China, and the virus rapidly spread throughout the country within 1–2 weeks. The high dependence of CKD patients on hemodialysis treatment and their intensive indoor treatment characteristics facilitated the sequential infection of all patients with COVID-19 within a week, thereby negating the impact of repeat infections on the study outcomes. Additionally, the timing of sample acquisition was more concentrated, which enhances the accuracy and informativeness of our findings.

The vWF plays a crucial role in the coagulation cascade, especially in COVID-19 patients [22, 23]. It is a large multimeric glycoprotein that is synthesized and secreted by endothelial cells and megakaryocytes. The vWF facilitates platelet adhesion and aggregation at sites of vascular injury by acting as a bridge between platelets and the exposed subendothelial collagen [24]. A deficiency or dysfunction of vWF can lead to von Willebrand disease, a bleeding disorder characterized by prolonged bleeding time and impaired platelet function [25]. In contrast, excessive vWF activity can contribute to thrombotic disorders such as deep vein thrombosis and pulmonary embolism [24, 26]. Previous clinical studies have demonstrated that COVID-19 leads to an increase in vWF levels in patients, with implications suggesting a potential association with vascular endothelial cell damage [27, 28]. Building upon these findings, the present study revealed a similar upregulation of vWF in CKD patients affected by COVID-19, indicating a link between the hypercoagulable state in these individuals and vWF. Notably, this study introduces the novel concept that elevated vWF levels play a crucial role in the COVID-19-induced hypercoagulability observed in CKD patients.

FBLN5 is a protein that belongs to the fibulin family of extracellular matrix proteins; however, its role in coagulation is not fully understood. Recent studies have suggested that FBLN5 may play a role in thrombosis and pulmonary embolism [29, 30]. FBLN5 has been shown to interact with vWF, and it may modulate the activity of vWF in the coagulation cascade and vascular injury [31, 32]. Additionally, FBLN5 is associated with the adhesion and aggregation of platelets [32–34], which are crucial events in the formation of a blood clot. Furthermore, FBLN5 has been implicated in the regulation of vascular smooth muscle cell proliferation and migration [34–36], which are processes that contribute to the development of atherosclerosis and thrombosis. Therefore, we suggest that COVID-19-induced thrombosis is associated with FBLN5. However, there is a lack of studies on the association between FBLN5 and thrombosis/coagulation, suggesting that upregulation of vWF/FBLN5 may be a novel mechanism for virus-induced thrombosis/coagulation.

One study investigation reported an intriguing outcome in FBLN-2 and FBLN-5 double knockout mice, carotid artery ligation injury resulted in vascular wall damage as well as the upregulation of vascular adhesion molecules, tissue factor expression and thrombosis [32]. Together with our results, these findings suggest that vWF/FBLN-5 disorder-induced thrombosis and vascular injury may be interrelated, or rather, thrombosis and vascular damage occur together.

Notably, our proteomic results do not entirely align with other COVID-19 proteomic findings, because we studied a population of CKD patients who commonly have cardiovascular disease complications. It has been reported that COVID-19 usually causes damage to the circulatory system with ACE2 being the main target. In CKD patients, COVID-19 may exacerbate the vascular injury and induce a hypercoagulable state by upregulating vWF/FBLN-5. These observations explain why our proteomic results differ from those obtained in non-CKD individuals and underscore the notion that CKD patients may be more susceptible to simultaneous vascular injury and thrombosis following COVID-19 infection.

There are several limitations to this study. First, the outbreak caused a sharp increase in infection rates due to the sudden COVID-19 policy decontrol in December 2022, which resulted in a significant number of healthcare workers and patients becoming infected concurrently. This caused a shortage of workforce and limited the amount of data that was available for recording. Second, data for many CKD patients were not recorded in the data due to transfer, death, or critical resuscitation, leading to potentially biased results during the statistical analysis. Third, further investigation is needed to determine the effects of gender, type of vascular access, and other complications on coagulation. Fourth, the limited sample size and restricted resources hindered the examination of primary renal diseases and related complications that could affect coagulation. Fifth, a comparable scenario was observed in patients undergoing peritoneal dialysis, where severe and critical patients were frequently transitioned to hemodialysis treatment or received a combination of both modalities. Due to the complexity of this situation, we did not include such patients in our statistical analysis. Additionally, the coagulation of the filters was not re-validated in vitro to assess the hypercoagulable state of the blood in this study. This decision was influenced by both the shortage of personnel and concerns about potentially delaying patient treatment. Last, the anticoagulant effect varies by type and usage of anticoagulant, which is a focus of future research. We conducted a retrospective analysis of our anticoagulation strategies and found that all CKD patients, except for those who died suddenly successfully completed their dialysis treatment. Additional studies will be published in the future.

## Conclusion

In conclusion, COVID-19 may upregulate the vWF/FBLN5 signaling pathway in the patients with severe/critical symptoms, thereby increasing the likelihood of extracorporeal coagulation during hemodialysis in CKD patients. These findings underscore the significant impact of the virus on the coagulation system of individuals with underlying kidney disease, emphasizing the necessity for continued research and vigilant monitoring in this population.

### Supplementary Information

**Supplementary Material 1.**

## Data Availability

All data generated or analyzed during this study are included in this published article [and its Supplementary information files]. (1) Figure S1. GO and KEGG analyses of upregulated proteins. (2) Figure S2. GO and KEGG analyses of downregulated proteins. (3) Supplementary 1. Raw data of the upregulated proteins. (4) Supplementary 2. Raw data of downregulated proteins. (5) Clinical results and raw data. (6) Raw proteomic data. The mass spectrometry proteomics data have been deposited to the ProteomeXchange via the iProX partner repository with the dataset identifier PXDO44377.

## Abbreviations

AKI: Acute kidney injury
CRRT: Continuous renal replacement therapy
CKD: Chronic kidney disease. COVID-19, coronavirus disease-2019
ESKD: End-stage kidney disease
HD: Hemodialysis
LWMH: Low molecular weight heparin
vWF: Von Willebrand factor
FBLN5: Fibulin-5
