# Supplementary material for: COVID-19 increases extracorporeal coagulation during hemodialysis associated with upregulation of vWF/FBLN5 signaling in patients with severe/critical symptoms
Source: BMC Infect Dis. 2024 Apr 22;24:427. doi: 10.1186/s12879-024-09245-9 (PMC11036607; doi:10.1186/s12879-024-09245-9)
Supplement: Supplementary file 1 — Supplementary Material 1. [file 12879_2024_9245_MOESM1_ESM.zip › Figure S1.pdf]

IGLV2-18

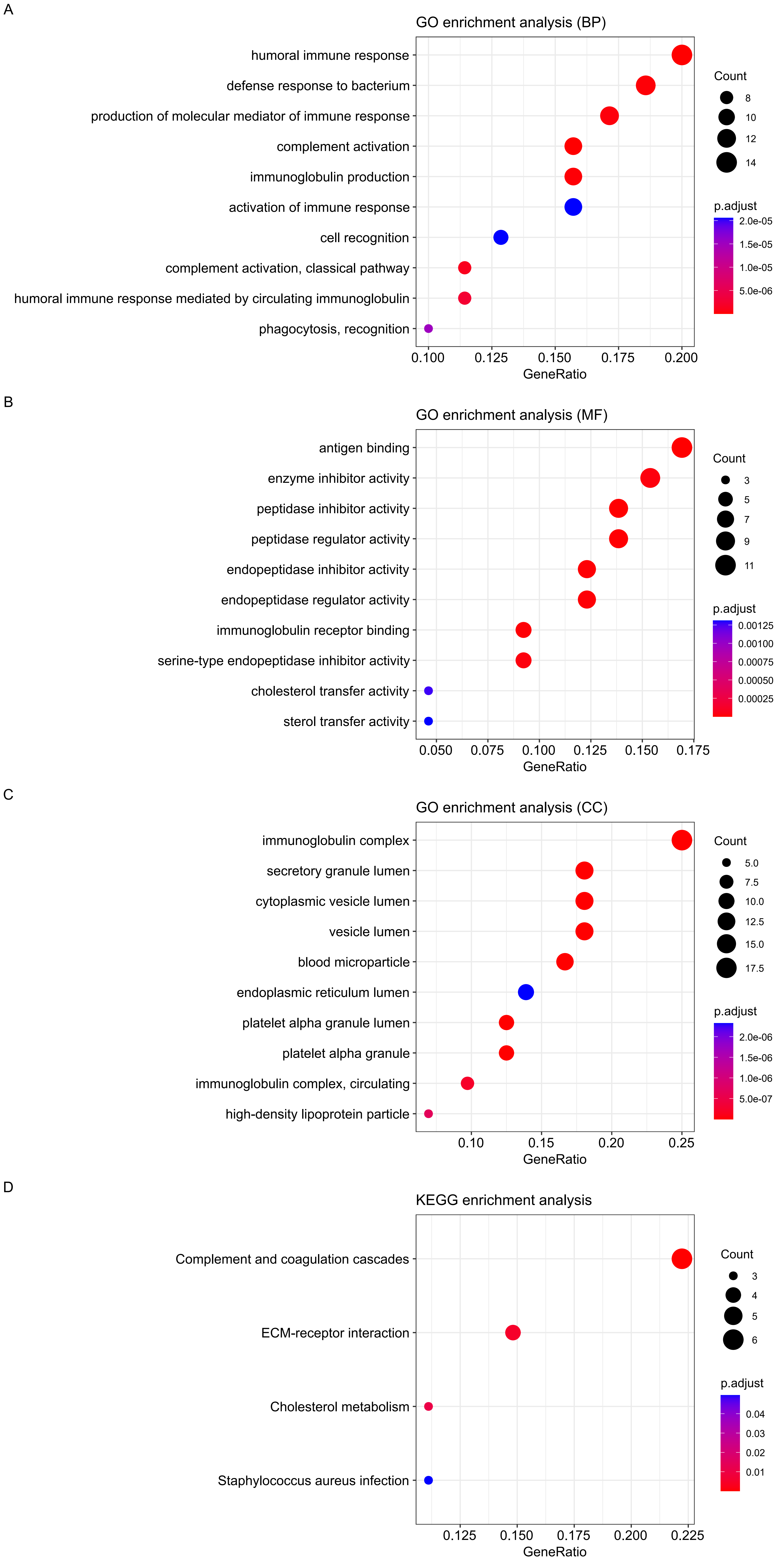

A
GO enrichment analysis (BP)
humoral immune response
defense response to bacterium
production of molecular mediator of immune response
complement activation
immunoglobulin production
activation of immune response
cell recognition
complement activation, classical pathway
humoral immune response mediated by circulating immunoglobulin
phagocytosis, recognition
0.100 0.125 0.150 0.175 0.200
GeneRatio
Count
8
10
12
14
p.adjust
2.0e-05
1.5e-05
1.0e-05
5.0e-06
B
GO enrichment analysis (MF)
antigen binding
enzyme inhibitor activity
peptidase inhibitor activity
peptidase regulator activity
endopeptidase inhibitor activity
endopeptidase regulator activity
immunoglobulin receptor binding
serine-type endopeptidase inhibitor activity
cholesterol transfer activity
sterol transfer activity
0.050 0.075 0.100 0.125 0.150 0.175
GeneRatio
Count
3
5
7
9
11
p.adjust
0.00125
0.00100
0.00075
0.00050
0.00025
C
GO enrichment analysis (CC)
immunoglobulin complex
secretory granule lumen
cytoplasmic vesicle lumen
vesicle lumen
blood microparticle
endoplasmic reticulum lumen
platelet alpha granule lumen
platelet alpha granule
immunoglobulin complex, circulating
high-density lipoprotein particle
0.10 0.15 0.20 0.25
GeneRatio
Count
5.0
7.5
10.0
12.5
15.0
17.5
p.adjust
2.0e-06
1.5e-06
1.0e-06
5.0e-07
D
KEGG enrichment analysis
Complement and coagulation cascades
ECM-receptor interaction
Cholesterol metabolism
Staphylococcus aureus infection
0.125 0.150 0.175 0.200 0.225
GeneRatio
Count
3
4
5
6
p.adjust
0.04
0.03
0.02
0.01

IGLV2-18

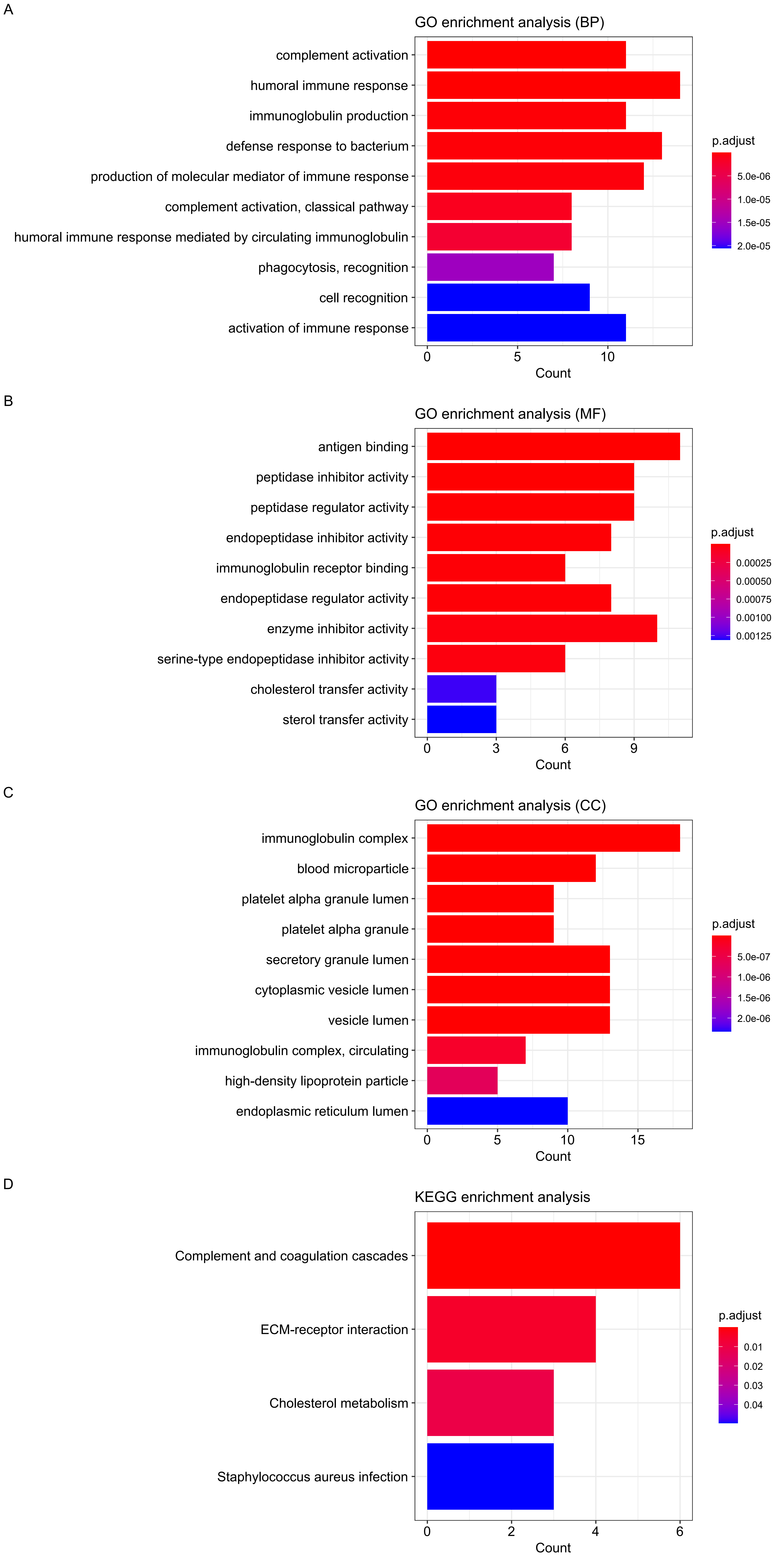

A
GO enrichment analysis (BP)
complement activation
humoral immune response
immunoglobulin production
defense response to bacterium
production of molecular mediator of immune response
complement activation, classical pathway
humoral immune response mediated by circulating immunoglobulin
phagocytosis, recognition
cell recognition
activation of immune response
0
5
10
Count
p.adjust
5.0e-06
1.0e-05
1.5e-05
2.0e-05
B
GO enrichment analysis (MF)
antigen binding
peptidase inhibitor activity
peptidase regulator activity
endopeptidase inhibitor activity
immunoglobulin receptor binding
endopeptidase regulator activity
enzyme inhibitor activity
serine-type endopeptidase inhibitor activity
cholesterol transfer activity
sterol transfer activity
0
3
6
9
Count
p.adjust
0.00025
0.00050
0.00075
0.00100
0.00125
C
GO enrichment analysis (CC)
immunoglobulin complex
blood microparticle
platelet alpha granule lumen
platelet alpha granule
secretory granule lumen
cytoplasmic vesicle lumen
vesicle lumen
immunoglobulin complex, circulating
high-density lipoprotein particle
endoplasmic reticulum lumen
0
5
10
15
Count
p.adjust
5.0e-07
1.0e-06
1.5e-06
2.0e-06
D
KEGG enrichment analysis
Complement and coagulation cascades
ECM-receptor interaction
Cholesterol metabolism
Staphylococcus aureus infection
0
2
4
6
Count
p.adjust
0.01
0.02
0.03
0.04

IGLV2-18

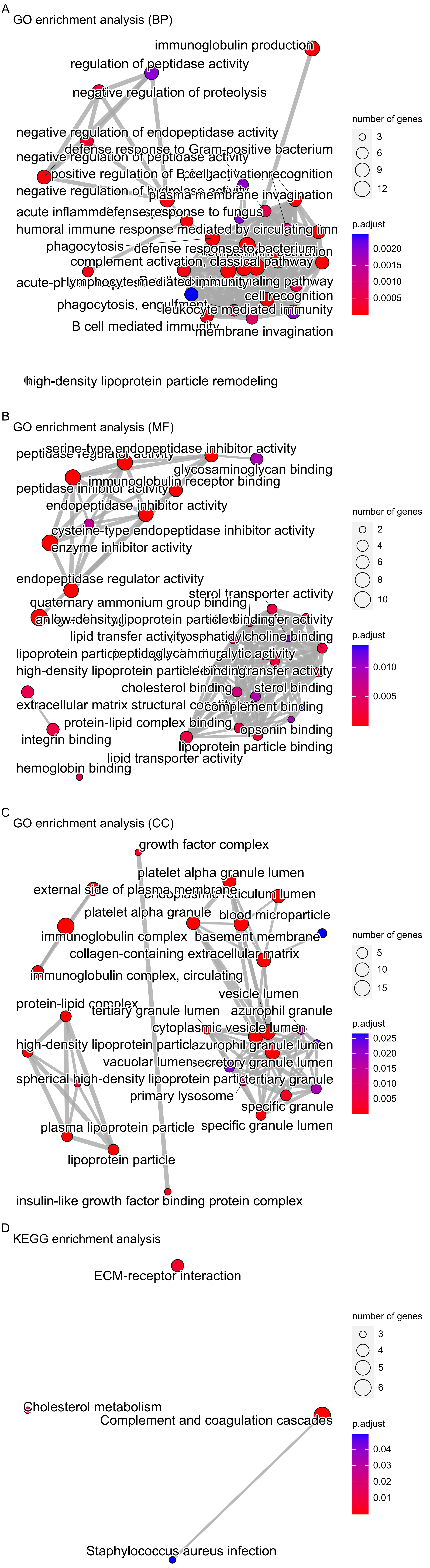
A
GO enrichment analysis (BP)
immunoglobulin production
regulation of peptidase activity
negative regulation of proteolysis
negative regulation of endopeptidase activity
defense response to Gram-positive bacterium
negative regulation of peptidase activity
positive regulation of B cell activation
recognition
plasma membrane invagination
response to fungus
humoral immune response mediated by circulating imm
phagocytosis
defense response to bacterium
complement activation, classical pathway
lymphocyte mediated immunity
signaling pathway
cell recognition
phagocytosis, engulfment
leukocyte mediated immunity
B cell mediated immunity
membrane invagination
high-density lipoprotein particle remodeling
number of genes
3
6
9
12
p.adjust
0.0020
0.0015
0.0010
0.0005
B
GO enrichment analysis (MF)
serine-type endopeptidase inhibitor activity
peptidase regulator activity
glycosaminoglycan binding
immunoglobulin receptor binding
peptidase inhibitor activity
endopeptidase inhibitor activity
cysteine-type endopeptidase inhibitor activity
enzyme inhibitor activity
endopeptidase regulator activity
sterol transporter activity
quaternary ammonium group binding
low-density lipoprotein particle binding
lipid transfer activity
phosphatidylcholine binding
peptidoglycan muralytic activity
high-density lipoprotein particle binding
transfer activity
cholesterol binding
sterol binding
complement binding
protein-lipid complex binding
integrin binding
opsonin binding
lipoprotein particle binding
lipid transporter activity
hemoglobin binding
number of genes
2
4
6
8
10
p.adjust
0.010
0.005
C
GO enrichment analysis (CC)
growth factor complex
platelet alpha granule lumen
external side of plasma membrane
reticulum lumen
platelet alpha granule
blood microparticle
immunoglobulin complex
basement membrane
collagen-containing extracellular matrix
immunoglobulin complex, circulating
vesicle lumen
protein-lipid complex
tertiary granule lumen
azurophil granule
cytoplasmic vesicle lumen
azurophil granule lumen
vacuolar lumen
secretory granule lumen
tertiary granule
primary lysosome
specific granule
plasma lipoprotein particle
specific granule lumen
lipoprotein particle
insulin-like growth factor binding protein complex
number of genes
5
10
15
p.adjust
0.025
0.020
0.015
0.010
0.005
D
KEGG enrichment analysis
ECM-receptor interaction
Cholesterol metabolism
Complement and coagulation cascades
Staphylococcus aureus infection
number of genes
3
4
5
6
p.adjust
0.04
0.03
0.02
0.01
