# Supplementary figures and images for: COVID-19 increases extracorporeal coagulation during hemodialysis associated with upregulation of vWF/FBLN5 signaling in patients with severe/critical symptoms
Source: BMC Infect Dis. 2024 Apr 22;24:427. doi: 10.1186/s12879-024-09245-9 (PMC11036607; doi:10.1186/s12879-024-09245-9)

A

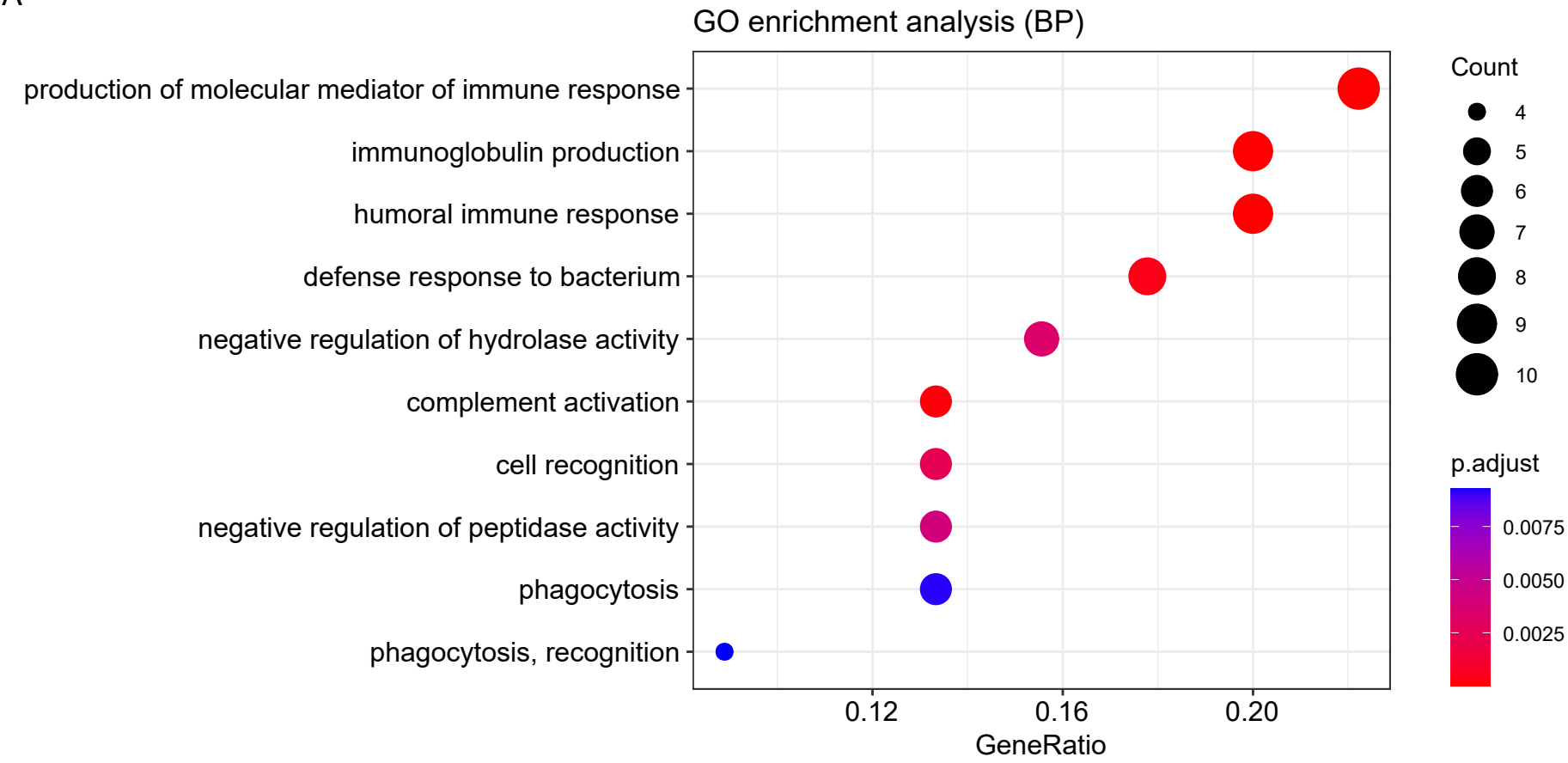

B

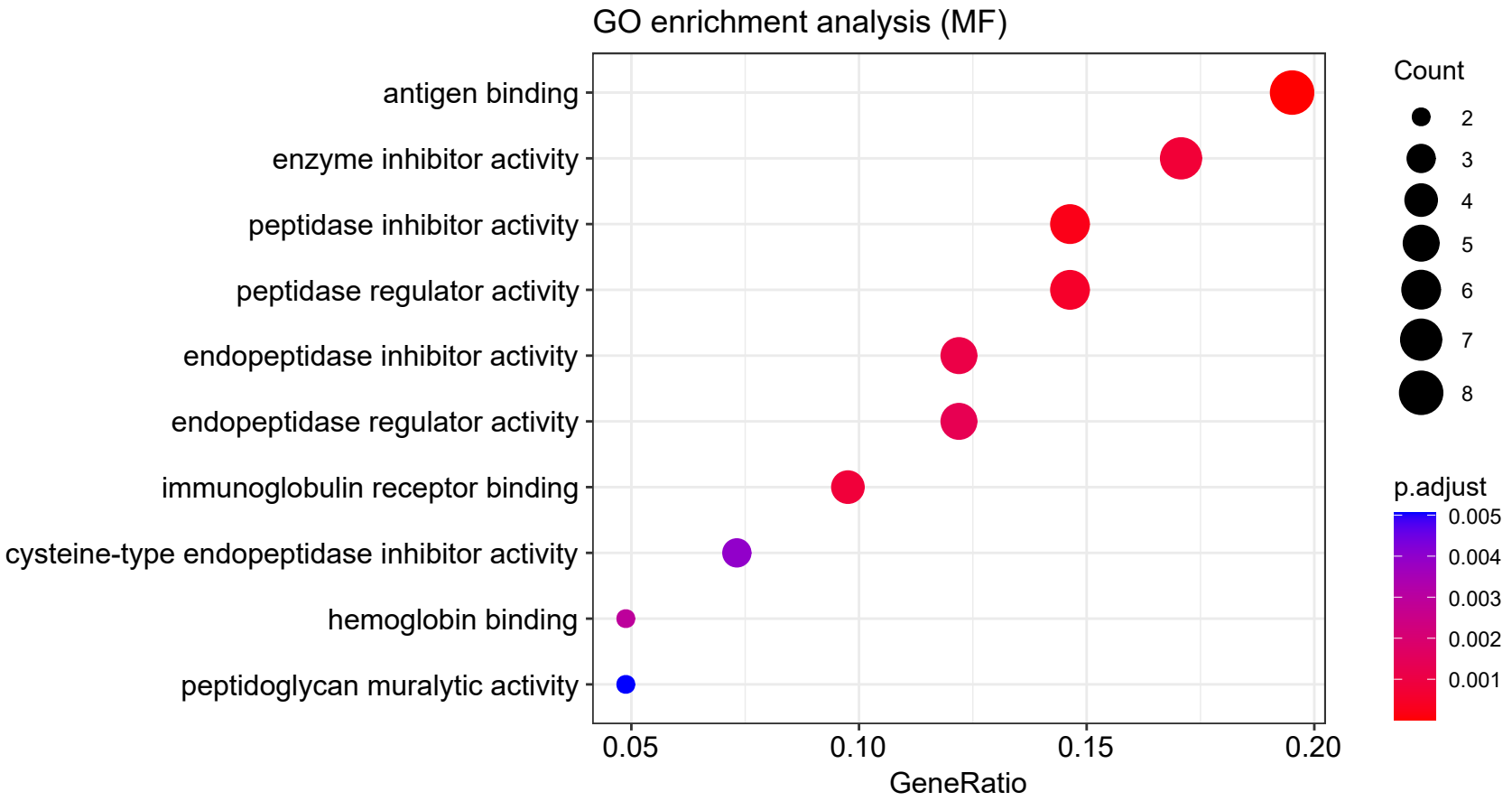

C

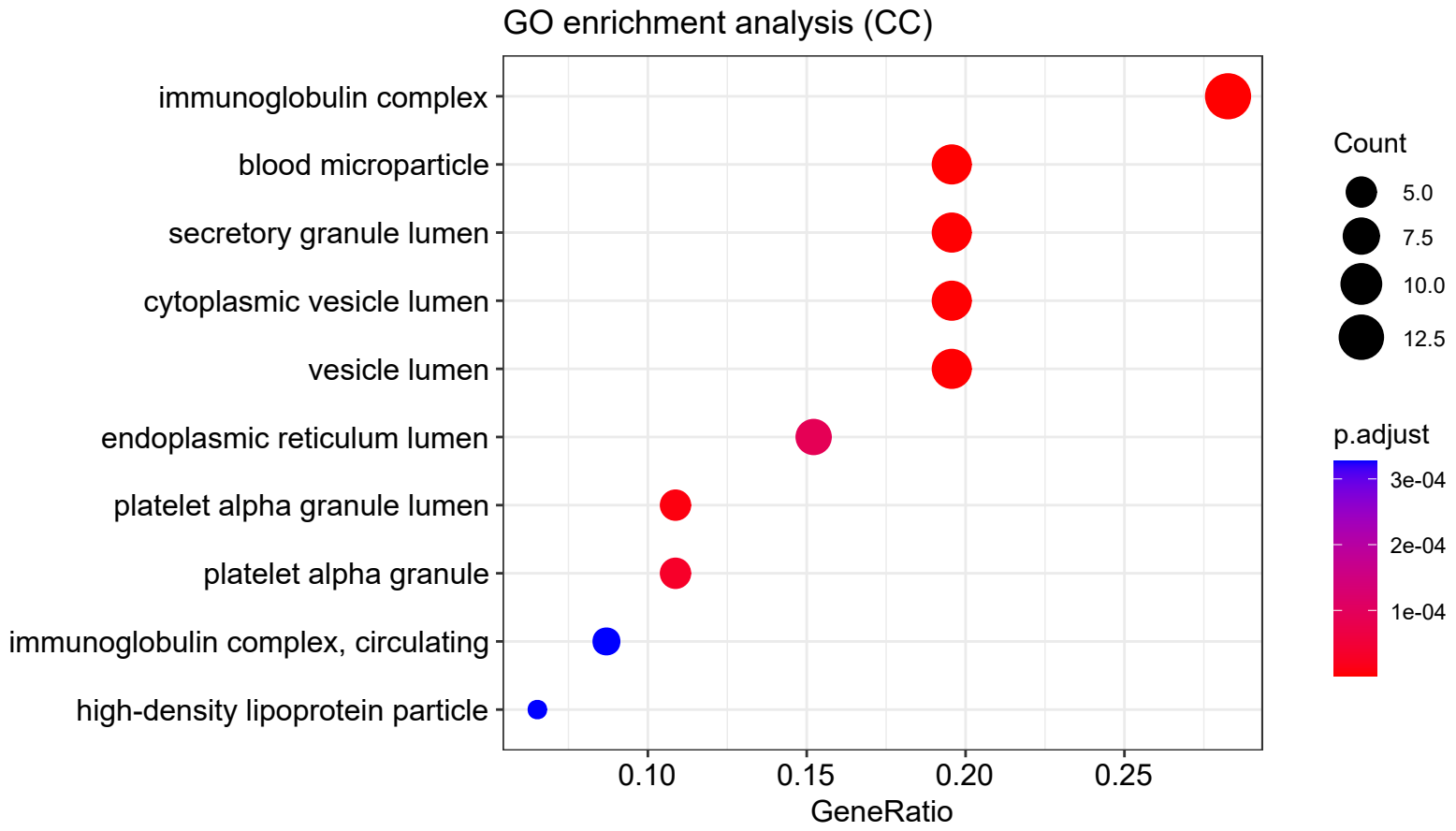

D

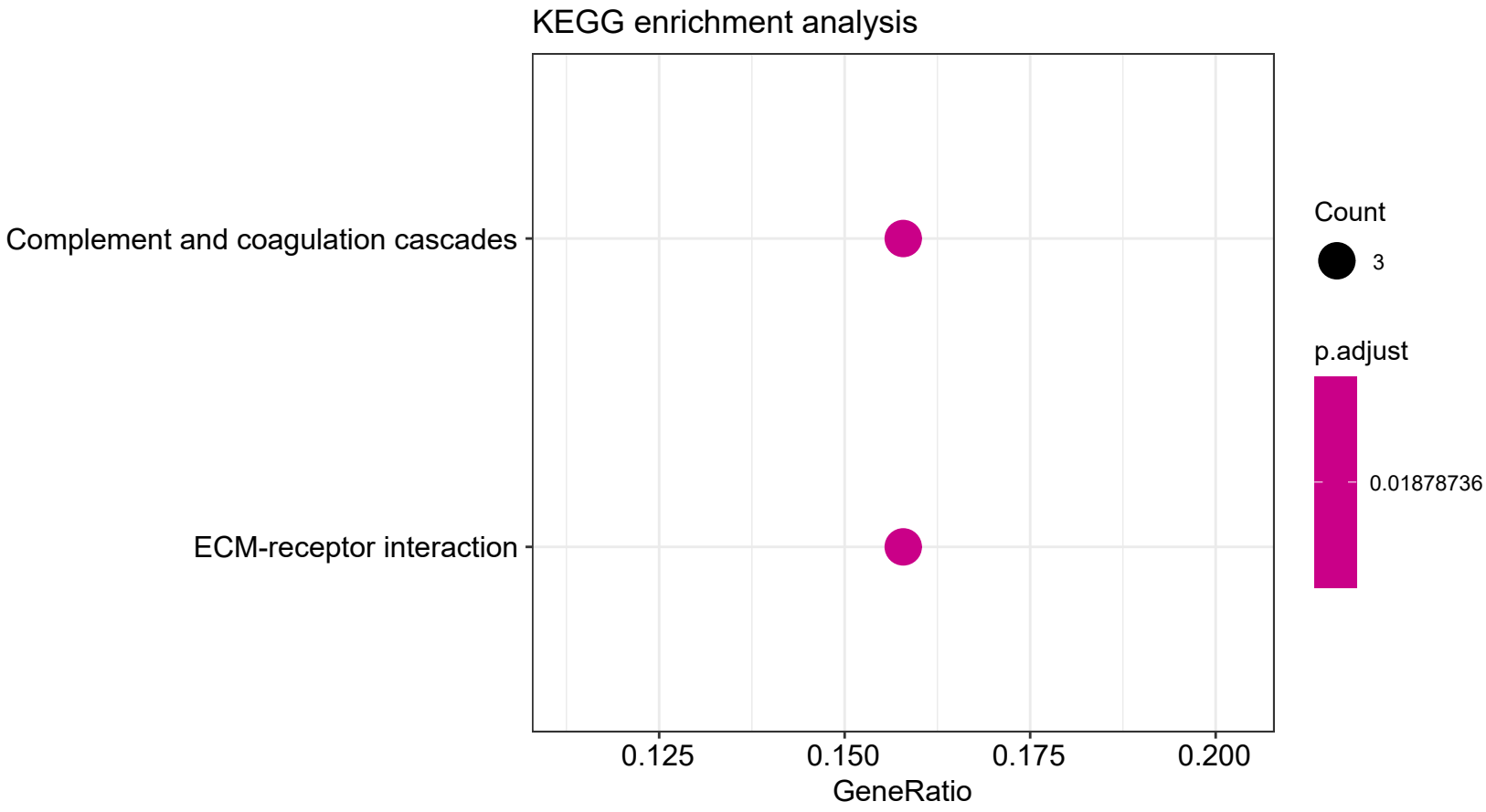

A

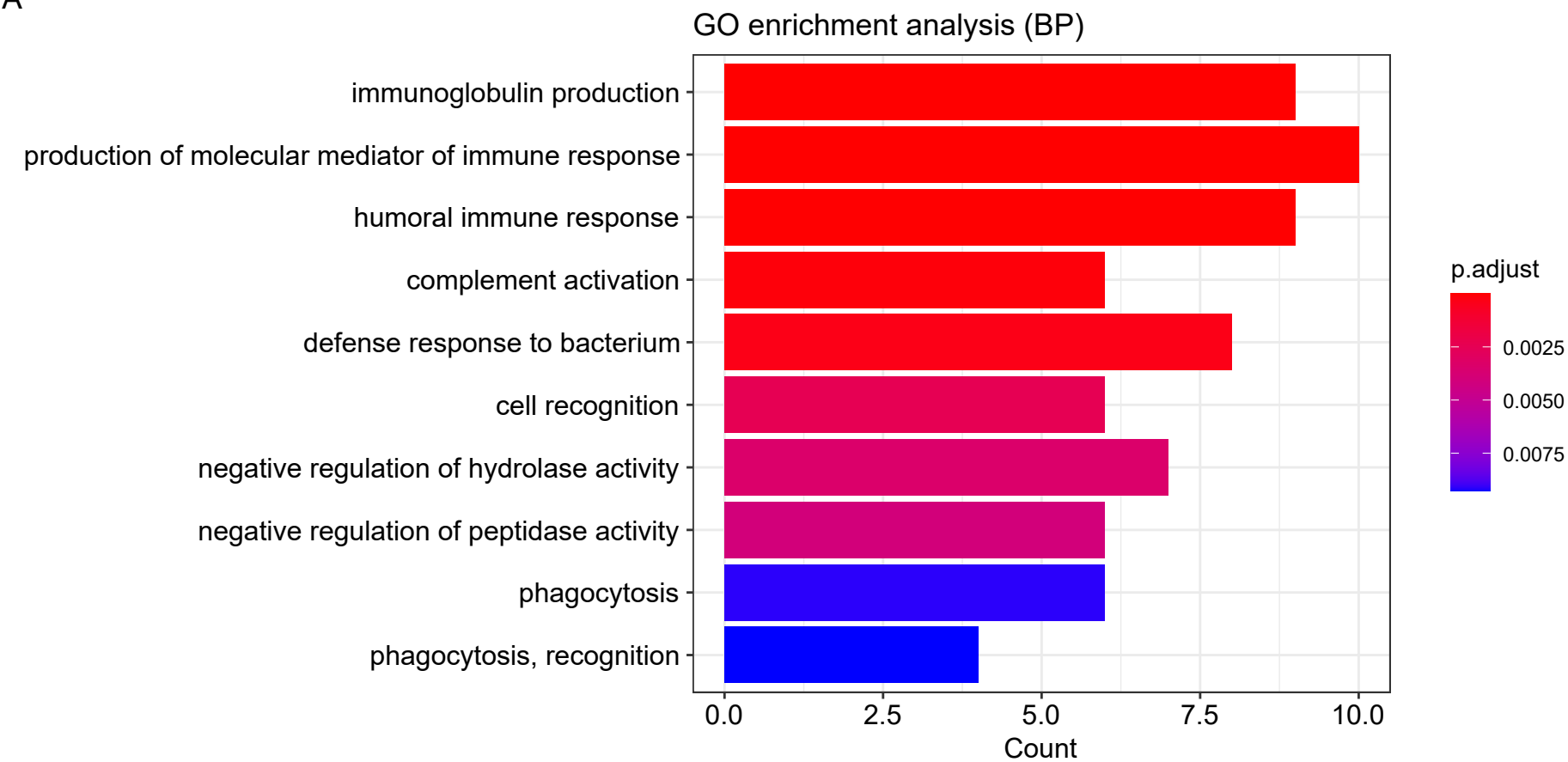

B

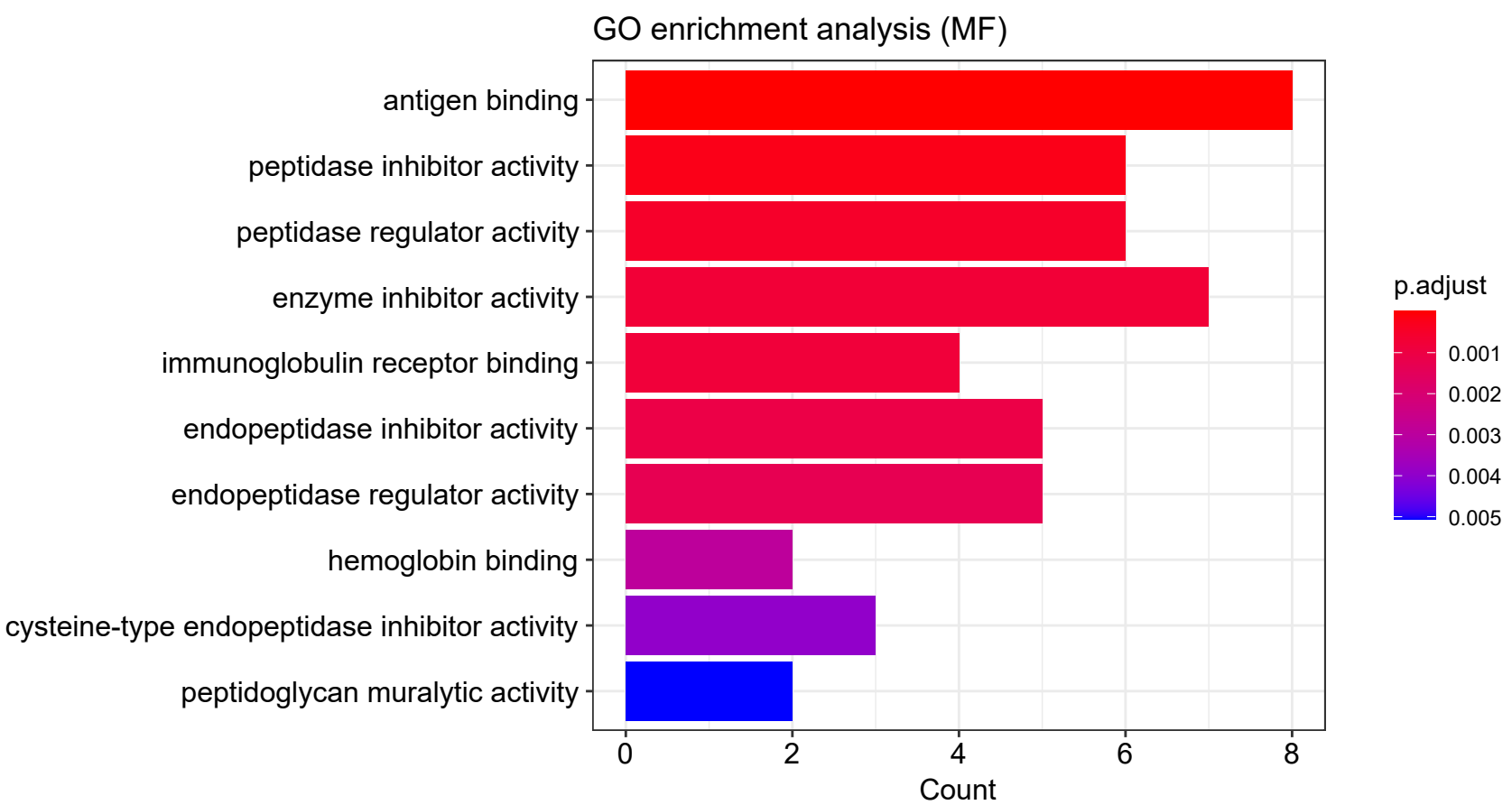

C

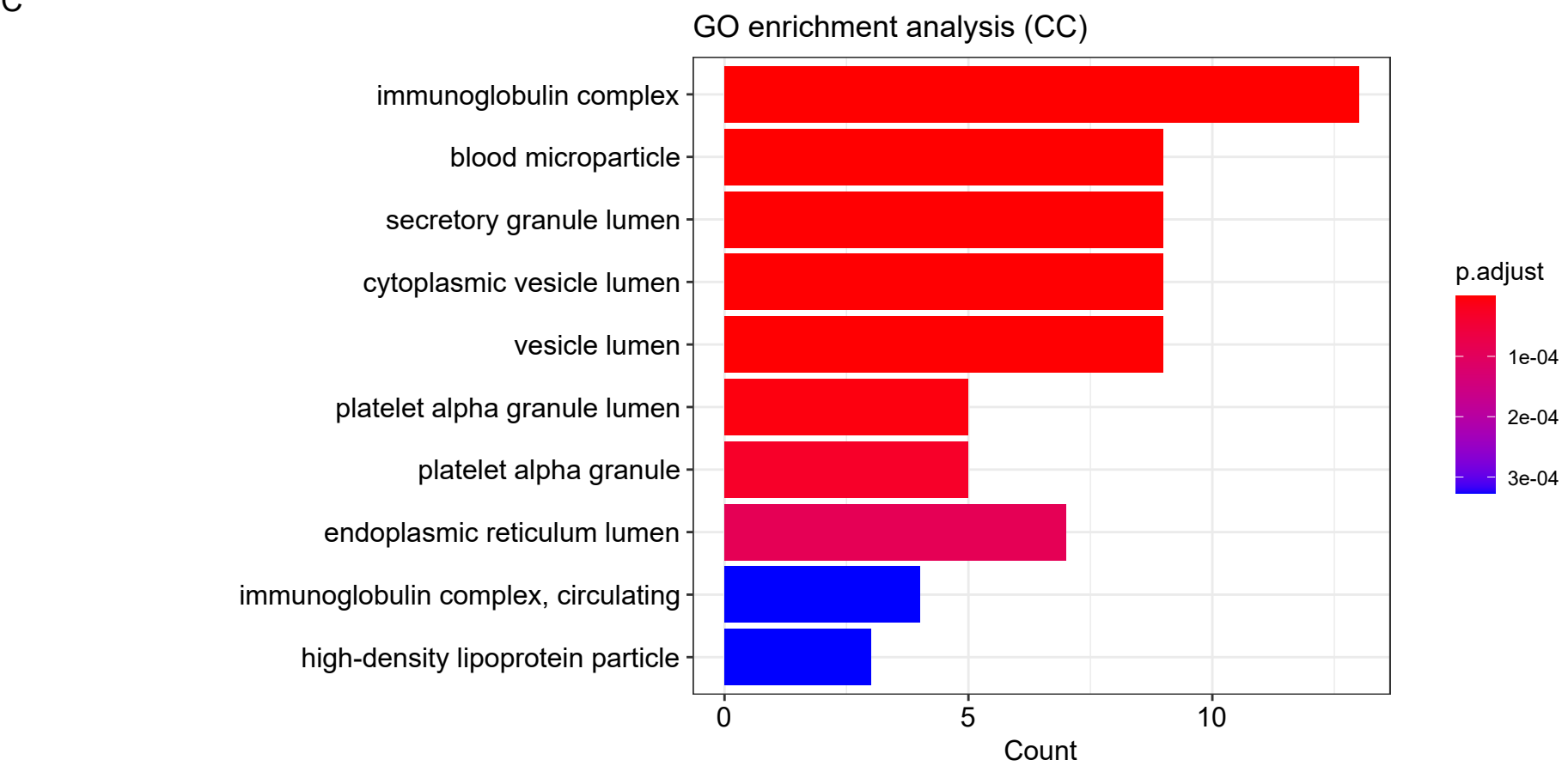

D

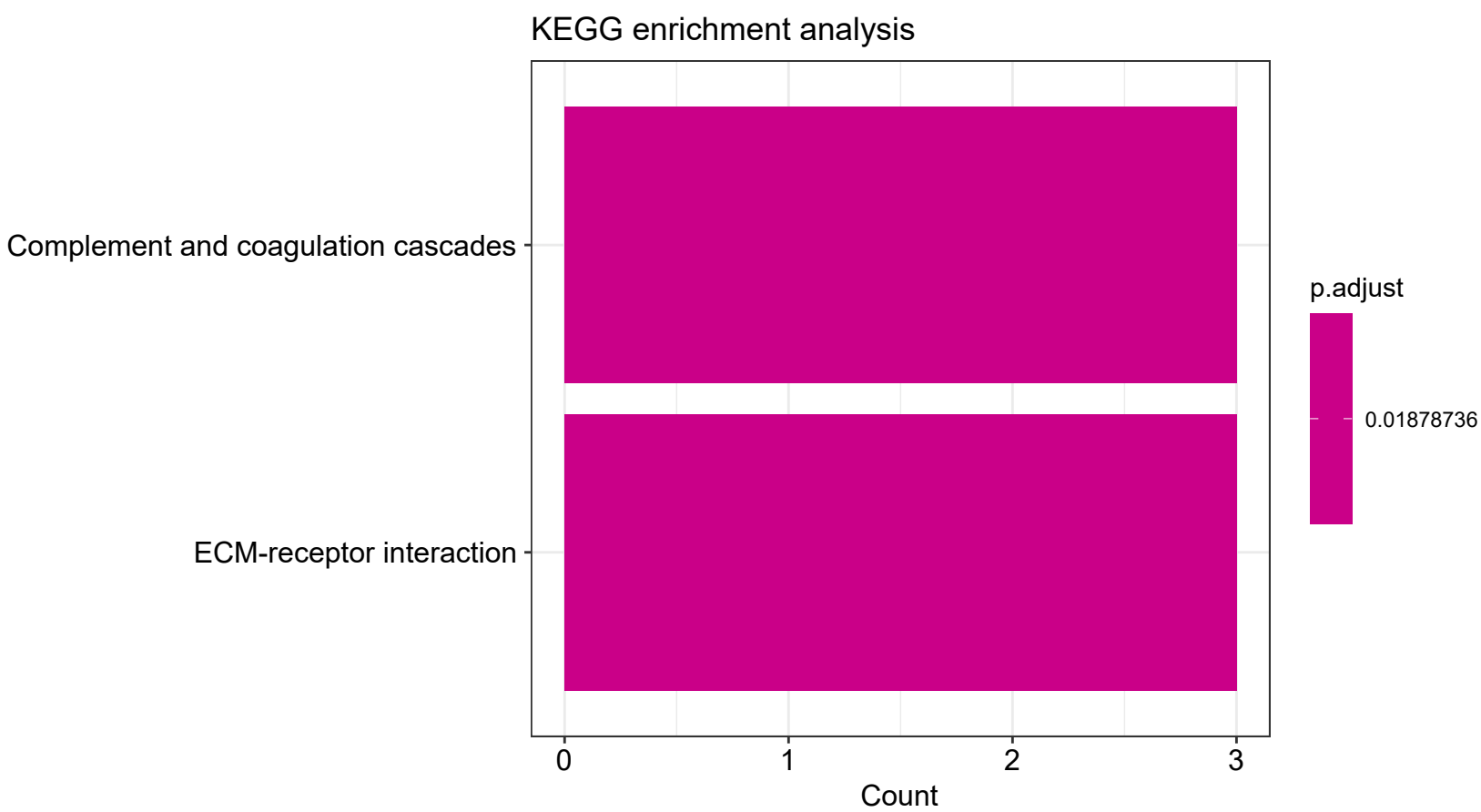

Supplement: Supplementary file 1 — Supplementary Material 1. [file 12879_2024_9245_MOESM1_ESM.zip › Figure S2.pdf]
